# Azurin: A Model to Study a Metal Coordination Sphere or Electron Transfer in Metalloproteins

**DOI:** 10.3390/ijms26094125

**Published:** 2025-04-26

**Authors:** Roman Tuzhilkin, Vladimír Ondruška, Miroslav Šulc

**Affiliations:** Department of Biochemistry, Faculty of Science, Charles University, Hlavova 2030, CZ-12843 Prague 2, Czech Republic; roman.tuzhilkin@natur.cuni.cz (R.T.); vladimir.ondruska83@gmail.com (V.O.)

**Keywords:** cupredoxin, azurin, redox properties, central metal/ion moiety, electron transfer

## Abstract

Azurin is a small blue copper protein that participates in redox reactions during anaerobic respiration in *Pseudomonas aeruginosa*, and there are a significant number of studies employing this model to investigate the electron transfer (ET) processes or coordination sphere of metal ion in metalloproteins. Azurin naturally contains Cu(II/I) as a central ion and is redox-active for a single electron ET. Moreover, azurin with no central ion (apo-azurin) is capable of binding other metal cofactors—e.g., Zn(II)—forming redox-inactive Zn-form and many others impacting the redox potential and structural variation in the active site’s arrangement. Also, mutations of amino acid residues in the immediate vicinity of the metal ion can influence the structure and functionality of a particular metalloprotein. Therefore, this review aims to summarize the abundant information about selected topics related to redox reactions and blue copper proteins, particularly azurin, and is structured as follows: (i) introduction to the structure, properties, and physiological role of this group of metalloproteins, (ii) the role of the equatorial and axial ligands of the central metal ions, or metal species, in the active site on the metal coordination sphere’s structure and related determination of the particular azurin form’s redox potentials, and (iii) the effects of the particular amino acid’s moiety (Phe, Tyr and Trp residues together with acceleration employing Trp-Trp π-π stacking interactions contrary to ET distance dependence) on the preferable type of long-range ET mechanism in an azurin-mediated model biomolecule. We assume that azurin is a suitable model to study the structural functionality of a particular central metal ion or individual amino acid residues in the central ion coordination sphere for studying the redox potential and ET reactions in metalloproteins.

## 1. Introduction

### 1.1. Redox Reactions Are One of the Cornerstones of Life

A complex system of reactions constantly occurs within cells with the aim of facilitating life. Chemical bonds are hydrolyzed and cleaved, entire functional groups are transferred between substrates, molecules are joined with newly formed covalent bonds and molecular isomers are inter-converted. Within this kaleidoscope of processes, an important vital role is played by redox reactions with a particular subset of electron transfer (ET) reactions. This group forms the core of energy metabolism pathways like the respiratory chain and photosynthesis, the crucial biochemical processes for life on Earth.

During the 20th century, multiple hypotheses were proposed to describe the mechanism of ET in proteins. According to some of them, electrons treat protein as a homogenous medium and do not follow any specific pathway through the structure. Others claimed that an electron travels through specific amino acids, cofactors and ligands in a strictly defined process with a designated route [1]. Currently, in the generally accepted view, the structure of a protein or protein complex has a serious impact on the ET process, and electrons travel usually through specific bonds in a molecule. The dawn of quantum physics in the middle of the 20th century and the discovery of wave–particle dualism introduced another dimension to the description of ET processes in biomolecules, with effects like electron or electron hole hopping and tunnelling. They helped to describe some very fast or long-range ET reactions (e.g., over 80−100 Å in the respiratory chain), which would not be possible in the realm of classical physics [2,3,4,5].

### 1.2. Blue Copper Proteins—Structure and Properties

The most important parameter for a redox reaction is its redox potential (E°′), which determines the capacity to convert one of the reaction partners to an oxidized state. In living systems, redox potentials fall within the physiological range defined by two fundamental reactions—the oxidation of water with E°′ approx. 1 V (measured against a standard hydrogen electrode, SHE; all potentials mentioned here are vs. SHE) and the reduction in protons to H_2_ with E°′ approx. –1 V [5]. In nature, just three groups of metalloproteins are enough to cover this ~2 V range of redox potential: the Fe-S proteins (E°′ from −700 to 500 mV), the cytochromes (E°′ from −500 to 350 mV) and the blue copper proteins (BCPs; E°′ from 100 to 800 mV) [6].

The latter received their name from the type-1 Cu complex (also known as “blue copper”, one of the six types of Cu complexes that occur in proteins [7]), which contains a single Cu ion, participates in single electron transfers and can also be found alone or with other Cu complexes (type 2, CuA) in some enzymes like ascorbate oxidase or nitrite reductase [8,9]. BCPs attracted attention and were used extensively as models to study the connection between the structures and properties of metal sites. Plastocyanin, rusticyanin and azurin are the most studied prominent representatives of the group.

**Figure 1:**
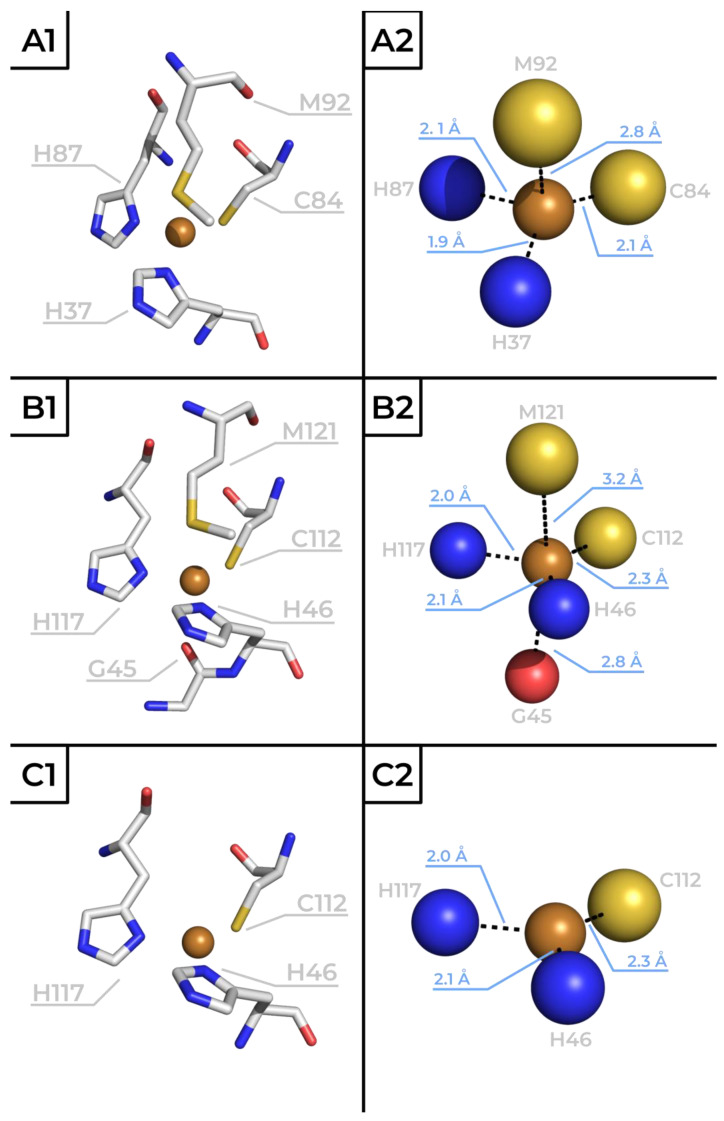
The diagrams of the primary coordination sphere of the copper atoms in two prominent BCPs: plastocyanin—Cu(II) (**A**), azurin—Cu(II) reduced at pH 5.5/9.0 (**B**) and oxidized—Cu(I) at pH 5.5 (**C**) states illustrated using stick (**1**) or bead (**2**) models. Bond distance values within the coordination sphere are mentioned in Å. The scheme was prepared by utilizing the PDB codes 1PLC [12], 4AZU [13] and 1E5Y [14]).

The Cu ion in the active site of BCPs is typically coordinated with three strong equatorial ligands—two imidazole ring N_ε_ atoms from two histidines’ residues and one S_γ_ atom of cysteine—and one weaker axial ligand, which is typically but not always the S_δ_ of methionine. In some cases, a second axial ligand is also present. This coordination sphere results in a distorted tetrahedral active site. Both Cu(II)-N_ε_(His) bonds are short (1.9–2.1 Å), while the Cu(II)-S_γ_(Cys) bond is slightly longer (2.1–2.3 Å) [4,10]; the length of the weaker axial Cu(II)-S_δ_(Met) bond is generally within the range of 2.8–3.1 Å [10]. The three-dimensional (3D) structures of the active sites with the particular values of the mentioned distances within the coordination sphere of two prominent BCPs, plastocyanin and azurin (in both redox states), are illustrated in Figure 1 (PDB codes 1PLC, 4AZU and 1E5Y).

**Figure 2:**
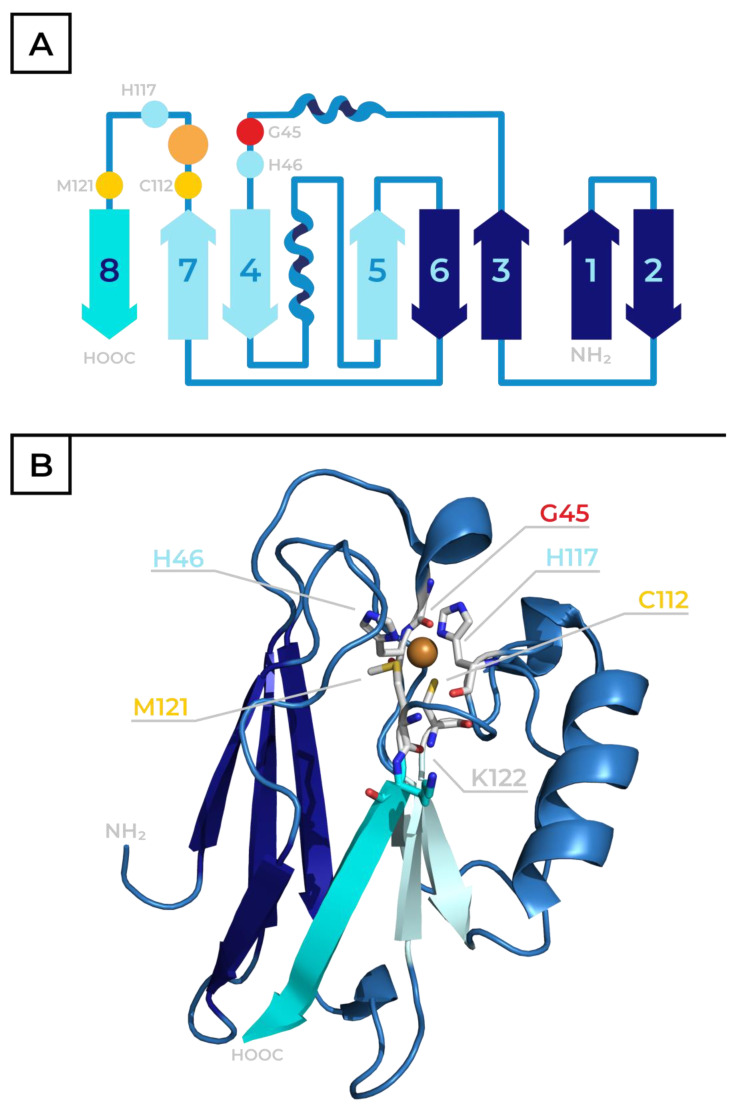
The secondary structure topology (A) and 3D structure (B) of azurin: (**A**) β-sheet structures are illustrated using arrows and numbered from the N-terminus (the two different configurations of the Greek key motive—powder/dark-colored blue); two α-helices are indicated by spirals and the loops are indicated by lines. The ligands (amino acid residues of the coordination sphere) and the copper ion are shown as colored spheres labelled with a one-letter amino acid residue code and its position in the azurin sequence; the central copper atom is illustrated by an orange sphere. The figure is adapted from the original work by Liu and coworkers (2014) [6]. (**B**) A 3D structure of azurin based on PDB code 4AZU: the central copper ion is shown as an orange sphere; the ligands (amino acid residues of the coordination sphere) are visualized as sticks labelled with a one-letter amino acid residue code and its position in azurin sequence; and the C-terminal β-sheet structure (number 8 from the N-terminus), which is responsible for the ET transfer, is highlighted using cyan together with Lys residue in position 122, illustrated as cyan sticks.

The primary structure of each BCP consists of 90–160 amino acids [10]. The 3D structure of plastocyanin was first solved by X-Ray and published in 1978 [11], followed by crystallography studies elucidating the structure of other BCPs. Typically, the secondary structure is dominated by eight β-sheets organized in a motif of the Greek key β-barrel. This 3D conformation can be found in plastocyanin and the previously mentioned enzymes containing type-1 Cu centers. In some cases, additional secondary structures like a second α-helix present in azurin (see simplified scheme and 3D protein structure in Figure 2) can be found in BCPs. Importantly, despite these minor differences, the available structural data show the similarity of the 3D structures in the vicinity of the central metal ion. The active sites of most BCPs share not only the general motif but also the subtle orientation and geometry of the participating residues [6].

## 2. Azurin—Physiological Role, Structure and Properties

Azurin is one of the most studied representatives of the BCP family. In nature, it is present in many bacterial species and acts as a single electron carrier in the anaerobic respiration [15] and deamination of primary amines [16]. Denitrification bacteria like *Pseudomonas* or *Alcaligenes* employ azurin as an electron carrier between the cytochrome c_551_ and nitrite reductase [17,18]. In the reaction catalyzed by this enzyme, nitrates and nitrites are reduced to N_2_ through nitrogen oxides (N_2_O, NO). The gaseous N_2_ is then released into the atmosphere, facilitating the nitrogen cycle in nature. This process generates the membrane proton gradient, which can power ATP pumps and provide energy to the bacteria.

Azurins are typically larger than plastocyanin, with a 120–130 amino acid sequence. The primary structure of azurin from *Pseudomonas aeruginosa* has 128 amino acids (see 3D protein structure in Figure 2B, PDB code 4AZU [13]). As in other BCPs, an eight-sheet Greek key β-barrel forms the core of the protein’s 3D structure. However, as mentioned earlier, this structure contains two α-helixes, unlike the more typical single α-helix configuration found in plastocyanin (see scheme in Figure 2A). The coordination sphere of the central Cu ion also deviates slightly from a typical type-1 Cu site. Instead of the deformed tetrahedral conformation (as present in plastocyanin, see Figure 1A), the metal ion interacts with five amino acid residues. His46, His117 and Cys112 function as equatorial ligands. Two axial positions are occupied by the sulfur of Met121 and the carbonyl oxygen of Gly45, resulting in a deformed trigonal bipyramid coordination sphere, as illustrated in Figure 1B. The structure of the azurin’s active site is dependent on its redox state, and the aforementioned coordination sphere is favored by the oxidized Cu(II) form. When the protein is reduced, the interactions between the Cu(I) cation and the axial ligands are weakened, and the active site assumes planar trigonal configuration (Figure 1C) [3,13,19].

In addition to this primary coordination sphere, a network of hydrogen bonds in the immediate proximity of the copper atom forms a secondary coordination sphere in azurin. These play a key role, as they can alter the properties of the active site by shielding it from water molecules or by raising the redox potential while simultaneously lowering reorganization energy for ET. To highlight one such interaction, in the vicinity of S_γ_ Cys112 (in direct interaction with Cu), a tetrahedral configuration is formed by two hydrogen bonds with backbone amide groups of Asn47 and Phe114 (located 3.6 Å and 3.4 Å away, respectively), together with S–Cu and S–C_β_ bonds. The electron density on the sulfur atom is altered due to the presence of these hydrogen bonds, which influences the properties of the S–Cu bond. More interactions of this nature can be found within the molecule, like the ones between Asn47 and carbonyl and the hydroxy group of Ser113, the interaction between His46 and Phe115 or even the His117–H_2_O molecule [6]. All of these have stabilizing or modulating effects on the active site.

The azurin sequence and the active site’s structure determine characteristic spectral properties similarly to other BCPs. In the ultraviolet–visible (UV-Vis) spectrum, two characteristic absorbance maxima at 280 nm and 630 nm (ε_280 nm_ ~9000 M^−1^ cm^−1^ and ε_630 nm_ ~5700 M^−1^ cm^−1^) [20,21] are present. The latter is attributed to the ${S(Cys)}_{\pi }\to Cu\left(II\right)d_{x^{2}-y^{2}}$ ligand-to-metal charge transfer transition [22], while a series of smaller absorption bands in the range of 650–1050 nm is caused by d→d ligand field transitions [23]. Because the absorbance at 280 nm arises from the presence of aromatic amino acids and is characteristic for protein moieties, the A_630_/A_280_ ratio can be used to determine the purity of azurin samples. For pure, wild-type *Pseudomonas aeruginosa* azurin (with one Trp, two Tyr and six Phe residues) this value is approx. 0.6 (0.58 ± 0.01 determined by van Kamp and coworkers [24]). When azurin accepts an electron and the copper ion is reduced to Cu(I), the absorption at 630 nm plummets as a result of the already-mentioned restructuring of the Cu’s coordination sphere (Figure 1B,C). This spectral property allows for the detection of an occurring ET reaction and is a major reason for azurin’s widespread application as a model in ET studies.

Additionally, the oxidation state of the Cu ion also affects the pH value of the isoelectric point (pI) of the particular protein. Azurin has a theoretical pI of 5.72 (computed by the ProtParam tool). The pI values determined experimentally for the Cu(II) form differ based on the measurement method and fall within the range of 5.4–5.7 [25,26,27]. However, in Cu(I), the reduced-form azurin’s pI value drops to 4.6 [24]. In the absence of the metal ion, the apo-form of azurin protein has a pI of 5.8 [24].

## 3. The Role of Metal and Amino Acid Residues in the Active Site of Azurin

The active site of azurin is highly conserved and stabilized around the central metal ion. However, it exhibits a high degree of malleability where individual residues acting as metal ligands can be altered to affect the redox properties without leading to the complete collapse of the structure. Furthermore, even the central copper ion itself can be substituted with other metals. This flexible nature has led to azurin being used as a model in numerous studies aimed at understanding the connections between the structure of metalloprotein active sites and their redox properties.

The Cu atom in azurin is substituted with other transition metals with similar atomic radius in the range of 1.35–1.40 Å ± 0.05 Å [28], like Ni [17], Co [29] or Fe [30]. Azurin is also metalated with heavy metals like Hg [31] (determined atomic radius 1.50 Å) [28]. Zn-azurin [32] also deserves a separate mention, as this form arises as a common contaminant during the azurin purification procedure. In those substitutions, despite the general structure remaining intact, reorganization occurs in the vicinity of the metal (Table 1). For example, in Zn-azurin the axial Met121 is no longer coordinated with the metal ion, while the other axial ligand—carbonyl oxygen of Gly45—moves approximately 0.03 nm closer to the zinc atom, resulting in a tetrahedral coordination sphere [32]. In Ni-azurin, both Cys112 and Gly45 move farther away from the metal. On the other hand, in Co form, ligand bonds with His46, His117 and Met121 become longer, while Gly45 shifts towards the metal. The structural changes result in different redox properties, where the nature of the change varies by each metal. The cyclic voltammetry with a pyrolytic graphite edge-plane electrode at pH 4.5 revealed that wild-type azurin has an almost 1 V lower redox potential when Cu(II) (E°′ = 310 mV) is replaced with Ni(II) (E°′ = –590 mV) [33]. The same trend is observed in azurin mutants metalated with Ni(II)-ion [5]. Although Ni(II) is a redox-active ion, azurin metalated with it cannot act as an electron donor or participate in ET [34]. When Fe(II) is introduced into wild-type azurin, it becomes highly resistant to oxidation to Fe(III), which is commonly found in biological systems, and the protein loses its redox properties [30]. However, a single mutation of Met121 for Glu residue restores the redox activity of Fe-azurin (E°′ = 320 mV) [33]. For the Zn(II) ion, the only oxidation state available is Zn(II), and Zn-azurin is consequently redox-inactive [35]. Because the mentioned metals occur at different abundances in living organisms (e.g., the concentrations in human organs are approximately: Fe >> Zn >> Cu >> Ni ≈ Co [36]), the Cu form in purified azurin preparations is not exclusive, and the presence of another metal ion, mainly the Zn form, is commonly detected [24].

Same as in the case of metal substitutions, site-directed mutagenesis can be used to modify individual amino acids in azurin with only minor effect on the overall structure but with a strong influence on redox properties of resulting mutants. A major role in fine-tuning of azurin’s redox potential is played by the two weak axial ligands of the central Cu(II/I) ion—Met121 and Gly45.

Typically, when the thioether of the Met, one of azurin’s axial ligands is replaced by a bulky hydrophobic residue (occupying a space like the Met residue) like Leu, Ile or Val, the redox potential of the protein increases by 100–140 mV. This effect can be attributed to hydrophobic side chains displacing other groups like water or electronegative ligands, which would otherwise stabilize Cu(II)’s state [38]. In contrast, highly polar residues (Asp or Glu) in position 121 stabilize the positive charge in the Cu(II) form of copper and reduce the overall redox potential by 50–120 mV [39]. These observations were corroborated by an extensive study by Garner et al. [40], in which unnatural methionine analogues were introduced into azurin to probe their effects on its redox potential, determined by a pyrolytic graphite edge electrode without the overlapping effects observed in less structurally similar natural amino acids. The axial Met121 was replaced with oxomethionine (E°′ = 222 mV), difluoromethionine (E°′ = 329 mV), selenomethionine (E°′ = 348 mV), trifluoromethionine (E°′ = 379 mV) and norleucine (E°′ = 449 mV). The analogues are mentioned in the order of increasing hydrophobicity, which strongly correlates with the increasing redox potential. Despite the resulting azurins covering the ~230 mV range of E°′, no major differences were observed in the protein’s structure (electron paramagnetic resonance data) or the UV-Vis spectra.

The role of the Gly45 carbonyl oxygen, the second azurin axial ligand which acts as a less typical axial ligand in the type 1 Cu center, can be illustrated by comparing the redox potential of azurin (E°′ = 310 mV determined by cyclic voltammetry with a pyrolitic graphite disk electrode at pH 7 and at 25 °C) to rusticyanin, where such an interaction is absent. Unlike other BCPs, rusticyanin’s redox potential lies significantly higher at 670 mV (at pH < 3 and an analogous experimental approach) [22]. It is proposed that the increased electron density around the Cu atom caused by the ionic interaction with the carbonyl oxygen of Gly in azurin stabilizes the Cu(II)’s state, lowering its redox potential [41].

Mutations in other positions typically only lead to modest changes in *P. aeruginosa* azurin’s redox potential, although a few amino acids in the previously mentioned secondary-coordination sphere of the active site serve as an exception. Modifying residues facilitating hydrogen bonding in the vicinity of the Cu center (Met44, Asn47, Trp48 or Phe114) allow us to predictably tune redox potential by altering the electron density on Cu ligands. For example, the Asn47Ser mutation results in a protein with ~130 mV higher redox potential than wild-type azurin because its effect leads to the strengthening of the hydrogen bonds to the Cys112 ligand from the primary Cu coordination sphere and the rigidification of the active site. Similarly, when Phe114 is substituted with Asn, a hydrogen bond with Gly45 is introduced into the structure, and the measured redox potential, determined using cyclic voltammetry with a glassy pyrolytic graphite electrode, increases by ~80 mV. On the other hand, the Phe114Pro mutation removes the direct hydrogen bond to Cys112, resulting in a lowering of the redox potential by ~90 mV, as determined with a pyrolytic graphite “edge” electrode [42].

Met121 axial ligand mutations, individual secondary coordination sphere mutations and metal substitutions all affect the final redox potential of azurin in distinct ways. Those distinct effects exhibit an additive nature, a property often used to reinforce and verify the findings presented earlier. In a study by Hosseinzadeh et al. [5], multiple modifications were cleverly combined to design azurin variants that cover the entire 2 V span of naturally occurring redox potentials. The highest potential of 970 mV was achieved by employing cyclic voltammetry with a pyrolytic graphite “edge” plane electrode at pH 5.0 in a 50 mM ammonium acetate buffer in the quintuple mutant Met44Phe/Asn47Ser/Phe114Asn/Gly116Phe/Met121Leu, thanks to the combined effect of highly hydrophobic Leu residue in position 121, hydrogen bond-strengthening Asn47 and Phe114 mutations and the introduction of two additional hydrophobic Phe residues into the secondary coordination sphere. On the other end of the redox potential scale, the lowest potential was achieved by introducing a highly polar Glu into the active site (azurin Met121Glu with E°′ = 184 mV) and consequently replacing the central Cu ion in the active site with the Ni ion, resulting in a redox potential of −945 mV, achieved by cyclic voltammetry in pH 8.0 phosphate buffer.

## 4. Azurin—ET Studies

BCPs importance goes beyond expanding our understanding of the connection between the structures and properties of metalloprotein’s active sites. A major part of our knowledge about the mechanisms of ET in proteins arises from studies on model BCPs like azurin and plastocyanin. In these experiments, a modified group, typically a transition metal complex, is introduced into the system to play the role of one of the redox partners (donor or acceptor) in the reaction. Here, we focus on the works of Gray and Winkler and their collaborators, which formed the basis of and later reinforced the ET hypotheses by determining the mechanisms and kinetics of the ET process in azurin.

The first mentioned study is related to investigation of the long-range ET mechanism in biomolecules together with a description of the structural differences and functional predispositions of selected amino acid residues as intermediates for multiple-step electron hopping or alternatively direct single-electron tunnelling. A rhenium complex, in which Re(I) is coordinated with three CO groups, a 4,7-dimethyl-1,10-phenantroline (dmp) and an imidazole nitrogen atom of a His sidechain located on the surface of a protein, is one of the systems employed to study ET in azurin. The metal complex acts as a photo-activatable electron donor, allowing the controlled initiation of the ET reaction. This study was performed with azurin mutants, introducing a His residue in position 124 (naturally taken by a Thr and located on the surface of the C-terminal β-sheet, visualized in cyan in Figure 2) to attach the Re complex. The position of Lys122, presented on the N-terminal’s initial part of the aforementioned C-terminal β-sheet, is substituted with one of three aromatic amino acids—Trp, Tyr or Phe—to study the ET between the Re donor and the Cu acceptor based on the nature of the bridging pathway in the vicinity of Met121, azurin’s axial ligand. In the crystal structure of one of the studied proteins (azurin mutant [Re(I)(CO)_3_(dmp)(His124)]Trp122, PDB entry 2I7O), the overall distance between the rhenium and the central copper ion was 19.4 Å, while the distance between the rhenium and indole of Trp122 was 8.9 Å, making it a suitable model system. The reorganization energy was assumed to be 0.8 eV. After the Re complex was activated, the excited electron was localized in the π-conjugated system of dmp, while Re(II)* was reduced by Trp122 and returned to the ground state, creating a cation radical Trp122^●+^, which was subsequently reduced by the Cu(I) in the active site, leading to its oxidation to Cu(II) [43]. This described reaction could be determined and its progress could be quantified by recorded time-resolved UV-Vis spectroscopy. The results show that the presence of Trp in position 122 allows for and favors an approximately 300× more rapid (time constant ~40 ns) two-step hopping ET process of Cu(I) → (Trp122)^●+^ → Re(II)* over the direct tunneling mechanism Cu(I) → Re(II)* between the metal sites observed in the other two mutants. For the Trp mutant, the calculated potential barrier between the donor and acceptor is 0.15 eV, and the free-energy gap for the first endergonic step of the transfer between Trp and Re(II)* is −0.03 eV. For distances over 20 Å, the hopping mechanism is predicted to only be possible when the free-energy changes for the endergonic intermediate steps are < 0.2 eV. On the other hand, when position 122 is occupied by Phe or Tyr, the energy gap between the bridging amino acid and Re(II)* is ~0.2 eV higher than for Trp, resulting in a much slower ET reaction that occurs through direct tunneling [44].

Azurin was also used to study distance dependence of ET along a β-strand [45]. For this purpose, a ruthenium complex, which functions on a similar principle to the previously described Re(I)(CO)_3_(dmp)(HisX), was used to modify the surface of the azurin C-terminal β-strand, with introduced accessible His residues at varying distances from the Cu in the active site. In the studied variants, the distance between the Cu(I) donor and the Ru(III) acceptor (R_DA_ in Å) was as follows: Ru(III)-His122-Cu(I) ~15.9; His82 ~16.9; His109 ~17.9; His124 ~20.6; His107 ~25.7 and His126 ~26.0. The measurements revealed a nearly perfect exponential distance dependence, with a decay constant (β) of 1.1 Å^−1^, a value similar to electron tunneling along saturated alkane bridges. Those data were later used to verify ab initio predictions of ET rates in proteins [46].

Another contribution of azurin to our understanding of ET processes is the exploration of the role of π-π interactions between aromatic amino acids. Gray and Winkler predicted the importance of aromatic side chains in ET in 2014 based on their lower ionization energies (Phe, 9.4 eV; Tyr, 8.5 eV; Trp, 7.8 eV) as compared with aliphatic amino acids (~9.6 eV) and their higher occurrence frequencies in the sequences of oxidoreductases as compared with other enzymes [3]. It is also known that efficient ET in DNA molecules is partially facilitated by π-π interactions between the aromatic bases inside of the structure. In 2019, Takematsu et al. used an azurin model to show that the same interactions can also play a major role in protein ET [47]. In their study, the Re-complex-modified azurin variant [Re(I)(CO)_3_(dmp)(His126)]Trp124Trp122 showed similar ET rates to the previously discussed [Re(I)(CO)_3_(dmp)(His124)]Trp122. Despite the overall distance between the Re and Cu increasing to 22.9 Å (PDB entry 6MJS), the time constant only increased slightly to ~70 ns but remained in the same order of magnitude. Moreover, no light-induced oxidation of Cu(I) was observed when either of the Tarps was replaced with Phe. The authors concluded that, while the presence of a single Trip enables a multi-step electron hopping mechanism and increases the ET rate by a factor of ~10^2^, two artificial Trps allow for a π-π stacking interaction, further increasing the factor to ~10^4^ as compared with a single-step electron tunneling mechanism.

To summarize the conclusions of the aforementioned azurin ET studies, the ability of long-range ET in biomolecules to occur through single-step direct tunnelling or multiple-step hopping mechanisms is affected by the following factors: (i) it is dependent on the electronic and structural parameters of a particular amino acid residue on the electron flow (hopping mechanism is dependent on the ability of the amino acid residue’s intermediate to stabilize a transiently localized electron or electron hole: Trp with high density on N1 indole π-radical Trp^●+^ >> Tyr and Phe [44,48]), (ii) the probability for single-step direct tunnelling has exponential distance dependence, with a decay constant (β) of 1.1 Å^−1^, and finally (iii) the π-π stacking interaction (e.g., two introduced Trp residues) accelerates the multi-step electron hopping mechanism.

## 5. Conclusions

Redox reactions, like photosynthesis and cellular respiration, are essential and play key roles for all living organisms. These reactions are facilitated by ET through a cascade of protein molecules and particular cofactors, a wide spectrum of which is utilized in nature. Due to their high variability and complexity, a detailed description of the mechanisms of naturally occurring ET reactions is challenging. Although in aqueous solution Cu(II/I)’s redox potential value is 153 mV, in biomolecules, particularly in model cupredoxin azurin, it can be tuned. With different types of central metal ions or amino acid residues in the metal coordination sphere azurin can cover a 2 V range of physiological redox potentials [5,39]. Interestingly, the metal substitutions structurally affect mainly azurin Met-S_δ_ axial ligand distance together with the second azurin axial ligand distance of Gly45 carbonyl oxygen (see Table 1). Similarly, the mutation of amino acid residue in position 121 has a major impact on azurin’s redox potential value (e.g., azurin vs. rusticyanin, Met121Leu/norLeu/…, Met121Glu) [5,22,39,40]. Additionally, other heteroatoms, except for the aforementioned sulfur atom in the position of the Met121 axial ligand in the amino acid residues in the metal coordination sphere, are important not only for the final value of the redox potential but also for the mechanism of the ET process occurring in the protein. The detailed hopping or tunneling mechanism is dependent on the total ET distance, the electronic and structural parameters of the particular amino acid residues on the electron flow trajectory (e.g., intermediate π-radical stabilization of a transiently localized electron or electron hole, or π-π stacking interactions) [44,47,48]. Therefore, expanding our understanding of these complex mechanisms should be considered as one of the major tasks of 21st-century chemistry and biochemistry. Azurin is a suitable model for studying the structural functionality of such electron transfer systems and for understanding the roles of individual amino acid residues or a particular central metal ion in the redox reactions of this group of metalloproteins.

## Figures and Tables

**Table 1:** The summary of bond distances between different central ion/metal (M and ligand atoms of residues within coordination sphere of azurin active site. The distance unit is Å. Calculated atomic radius of central ion [37], values of pH in the isoelectric point and PDB codes are mentioned. Adapted and modified [4]. Bonds with increased distance values (in comparison to the Cu(II) azurin) are highlighted in italics, while particularly significant increases are also bolded. Bonds in which the distance values are noticeably reduced are underlined.

Azurin (*Metal*)	pI	Calculated M Atomic Radius	M-N_ε_ (His46)	M-S_γ_ (Cys112)	M-N_ε_ (His117)	M-S_δ_ (Met121)	M-O (Gly45)	PDB
**Cu(II)**	5.5	1.45	2.11	2.27	1.99	3.18	2.84	4AZU
**Cu(I)**	4.6	-	2.05	2.30	1.98	3.16	***3.11***	1E5Y
**Ni(II)**	5.7	1.49	2.15	*2.49*	2.07	***3.34***	***3.35***	1NZR
**Co(II)**	5.7	1.52	***2.39***	2.34	2.27	***3.56***	2.23	1VLX
**Zn(II)**	5.7	1.42	2.07	2.30	2.01	***3.38***	2.32	1E67

## Data Availability

No new data were created or analyzed in this study. Data sharing is not applicable to this article.
